# Fetal Genotyping in Maternal Blood by Digital PCR: Towards NIPD of Monogenic Disorders Independently of Parental Origin

**DOI:** 10.1371/journal.pone.0153258

**Published:** 2016-04-14

**Authors:** Sara Perlado, Ana Bustamante-Aragonés, Marta Donas, Isabel Lorda-Sánchez, Javier Plaza, Marta Rodríguez de Alba

**Affiliations:** 1 Department of Genetics, IIS-Fundación Jiménez Díaz UAM, CIBERER, Madrid, Spain; 2 Department of Obstetrics & Gynecology, Fundación Jiménez Díaz-IIS, Madrid, Spain; Odense University Hospital, DENMARK

## Abstract

**Purpose:**

To date, non-invasive prenatal diagnosis (NIPD) of monogenic disorders has been limited to cases with a paternal origin. This work shows a validation study of the Droplet Digital PCR (ddPCR) technology for analysis of both paternally and maternally inherited fetal alleles. For the purpose, single nucleotide polymorphisms (SNPs) were studied with the only intention to mimic monogenic disorders.

**Methods:**

NIPD SNP genotyping was performed by ddPCR in 55 maternal plasma samples. In 19 out of 55 cases, inheritance of the paternal allele was determined by presence/absence criteria. In the remaining 36, determination of the maternally inherited fetal allele was performed by relative mutation dosage (RMD) analysis.

**Results:**

ddPCR exhibited 100% accuracy for detection of paternal alleles. For diagnosis of fetal alleles with maternal origin by RMD analysis, the technology showed an accuracy of 96%. Twenty-nine out of 36 were correctly diagnosed. There was one FP and six maternal plasma samples that could not be diagnosed.

**Discussion:**

In this study, ddPCR has shown to be capable to detect both paternal and maternal fetal alleles in maternal plasma. This represents a step forward towards the introduction of NIPD for all pregnancies independently of the parental origin of the disease.

## Introduction

Monogenic diseases result from mutations in a single gene. Although relatively rare, these disorders affect millions of people worldwide. The global prevalence of all single-gene diseases at birth is approximately 10/1000 [1]. In spite of the low prevalence, the number of cases worldwide must not be underestimated.

At present, prenatal diagnosis (PD) is available for pregnancies at risk of a monogenic disease. Conventional prenatal genetic diagnosis entails the collection of a fetal tissue sample by invasive obstetric methods (chorion villus sampling/amniocentesis), which based on recent studies has an associated risk of fetal loss of 0–1% [2, 3]. The analysis of circulating cell-free fetal DNA (ccffDNA) in maternal blood [4] allows for non-invasive prenatal diagnosis (NIPD) of fetal genetic disorders to be performed somewhat more safely and without the need for a trained Obstetrician, since it only requires a venipuncture. The NIPD studies currently used in clinical practice are fetal sex determination [5], fetal RhD determination [6], a limited number of monogenic diseases with a paternal origin [7, 8] and screening for the most common aneuploidies [9–12].

Because of the coexistence of maternal and fetal DNA in the maternal plasma sample, NIPD approaches have been mainly limited to the study of paternally inherited or *de novo* alleles that are not present in the maternal genome, as the presence of these alleles in the maternal plasma ensures that they originated in the fetus and can be associated with the fetal genome, while absence of these alleles indicates a non-carrier fetus. Following this presence/absence criterion, NIPD of different monogenic disorders using different technologies has been reported in the literature [13–16].

Recently, NGS (Next-Generation Sequencing) panels for the analysis of different mutations associated with cystic fibrosis and skeletal dysplasias have been incorporated into routine clinical practice [7, 8]. These panels are being offered to pregnancies with sonographic findings associated to these diseases or in which the father is carrier of one of the mutations included in the panel.

The application of more sensitive technologies like NGS and digital PCR (dPCR) is opening the NIPD field to the analysis of maternally inherited fetal alleles. Digital PCR [17] allows precise allele quantification in maternal plasma and its relative dosage (Relative Mutation Dosage, RMD) [18]. As a result of the RMD calculation either a balanced or imbalanced allelic ratio is obtained and correlated with a fetal genotype.

The present study shows a validation analysis of the digital PCR technology for fetal allele detection and RMD in maternal blood. The aim was to evaluate the potential of ddPCR for NIPD studies of fetal mutations independently of their parental origin. For this purpose, a Single Nucleotide Polymorphism (SNP) validation strategy was used to mimic the inheritance pattern of monogenic disorders (autosomal dominant and recessive diseases and X-linked disorders). None of the SNPs used were disease associated.

## Material and Methods

### Study design

**Fig 1:**
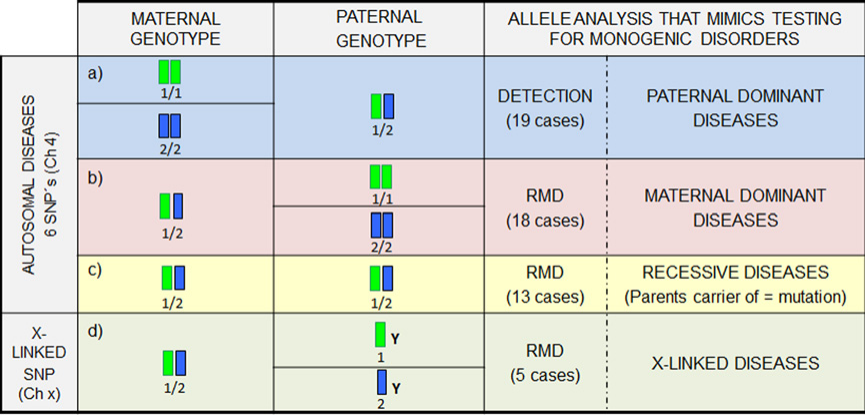
Schematic representation of the study design based on a SNP study showing: parental genotypes, the data analysis approach in maternal plasma and the inheritance pattern mimicked. Parental genotyping combination for allele 1/allele 2 of a certain SNP can be used to represent an inheritance pattern for a point mutation. The different parental genotype combinations and their correspondence with a specific inheritance pattern are detailed in the figure. Analysis of exclusive paternal sequences requires a detection approach while the study of maternal genomic regions requires a Relative Mutation Dosage (RMD) analysis. A simulation of an NIPD for a dominant disease with paternal origin can be done using a case in which the mother is homozygous for an SNP and the father is heterozygous (blue background). Presence/absence of the exclusive paternal allele could be associated with a fetal genotype. On the contrary, a heterozygous mother and a homozygous father will mimic a dominant disorder with a maternal origin (red background). This simulation can be also considered for recessive diseases in which both parents are carriers of a different mutation. NIPD for a recessive disease in which both parents are carriers of the same mutation can be simulated by taking as an example a couple in which the mother and the father are heterozygous for an SNP (yellow background). Finally, the simulation of NIPD for X-linked disorders can be done by a case in which mother is heterozygous for a SNP and the father is hemizygous (green background).

Because of the low prevalence of rare diseases, the study was not carried out by the analysis of real pathogenic variants but with the analysis of SNPs, in a way that they were mimicking different inheritance patterns. For the purpose, 15 family quartets (mother, father, CVS and maternal plasma) were selected for this work. First, parents and CVS were genotyped for 7 SNPs (6 autosomal and 1 on chromosome X). Once genotyped, only in those family quartets in which parental genotypes for an specific SNP allowed us to simulate an inheritance pattern, fetal genotyping in the maternal plasma samples was performed (Fig 1). Presence of fetal DNA in maternal plasma was assumed in case of detection of exclusive paternal alleles or an imbalanced allelic ratio. For those cases in which a balanced allelic ratio was detected, the presence of fetal DNA was confirmed by detection of the *SRY* gene (male pregnancies) and *RASSF1A* gene (female pregnancies).

### Subject recruitment and Sample collection

Once this study was revised and approved by the Ethics Committee of the Fundación Jiménez Díaz Hospital, patient recruitment was performed. Three inclusion criteria were considered: a) possibility of blood collection from both members of the couple (during subject recruitment, all couples provided written informed consent in accordance with the Declaration of Helsinki); b) availability of the prenatal sample (chorionic villus sample (CVS) or amniotic fluid (AF)) for confirmation of plasma results; and c) gestational age at time of sample collection between 8 and 20 weeks.

Blood samples were collected from 15 couples (20 cc of blood in EDTA tubes) who visited the Obstetrics and Gynecology Unit at Fundación Jiménez Díaz Hospital for a prenatal invasive procedure.

### Sample processing and DNA extraction

Maternal blood samples were processed as previously described [5]. Total DNA was extracted from 1 ml of plasma using the MagNaPure Compact Nucleic Acid Isolation Kit I-Large Volume kit and the Total NA Plasma 1000 v3 protocol of the MagNaPure Compact (Roche, Mannheim, Germany). Final elution volume was 50μl.

Control DNAs included paternal genomic DNA (pDNA) and maternal genomic DNA (mDNA) obtained from peripheral blood and fetal DNA (fDNA) obtained from CVS or AF. Genomic pDNA and mDNA were extracted from 1 ml of peripheral blood using the MagNaPure Compact (Roche, Mannheim, Germany) and eluted in 200 μl. Fetal genomic DNA samples were extracted with the EZ1 Robot (QIAGEN, Hilden, Germany) according to the manufacturer’s protocol.

### SNP Genotyping by Real-Time PCR

In order to select informative SNPs to be studied in maternal plasma samples, six SNPs located on chromosome 4 (rs4974667, rs4874668, rs798750, rs762837, rs612973, and rs2236995) were studied in 13 couples. Additionally, one SNP on chromosome X (rs1128363) was studied in five couples.

SNPs were studied by the use of pre-designed Taqman assay primer/probe set (Life Technologies) in which allele 1 is labelled in VIC and allele 2 is labelled in FAM.

Parental genomic DNAs in each couple were quantified and diluted to a final concentration of 20 ng/μl. PCR reaction had a final volume of 25 μl, including 1.25 μl of 20X Taqman Genotyping assay (Life Technologies, Carlsbad, CA); 12.5 μl of Taqman Genotyping Mastermix; 1 μl of genomic DNA (20 ng/μl); and 10.25 μl of water. Two replicates were performed per sample in all cases. PCR was carried out in the Step One Real-Time PCR System (Life Technologies, Carlsbad, CA).

An SNP was considered as informative when only the paternal genotype, maternal genotype, or both were heterozygous for that specific nucleotide (Fig 1).

### Fetal genotyping by digital PCR from maternal plasma

Using Droplet Digital PCR (ddPCR) technology, the sample was partitioned into 20,000 nanoliter-sized droplets. This partitioning enables the measurement of thousands of independent amplification events within a single sample.

The ddPCR reaction mix included 11 μl of *ddPCR Mastermix no dUTP for probes* (Bio-Rad, Pleasanton, CA), 0.55 μl of each Taqman assay (Life Technologies, Carlsbad, CA), and 10,45 μl for plasma DNA (4–5 replicates), and 1 μl of genomic DNAs (pDNA, mDNA, and fDNA samples) (two replicates). PCR final volume was 22 μl. Cartridges (DG8 Cartridges, Bio-Rad, Pleasanton, CA) with the ddPCR reaction mix and specific oil (Droplet Generator Oil, Bio-Rad, Pleasanton, CA) were inserted into the QX200 droplet generator (Bio-Rad, Pleasanton, CA), which is used to partition ddPCR reaction mix into 20,000 nanoliter-sized droplets.

**Fig 2:**
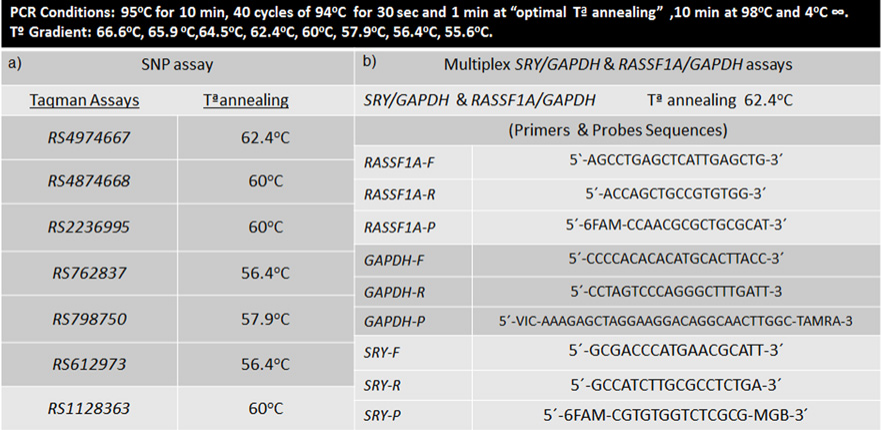
Primer and probe sequences and ddPCR conditions. This figure also includes optimal Temperature of Annealing (Ta) for each Taqman assay for the multiplex *SRY/GAPDH* and *RASSSF1A/GAPDH* assays.

PCR was carried out in a C1000 Touch Thermal Cycler (Bio-Rad, Pleasanton, CA), following the conditions shown in (Fig 2) and the PCR product was analyzed individually in the QX200 droplet reader. In this procedure, droplets pass through a two-color optical detection system in a serial manner. The PCR-positive and PCR-negative droplets are counted to provide absolute quantification of target DNA in digital form.

A previous temperature gradient was performed in order to select the optimal temperature for each primer/probe SNP Taqman assay (Fig 2). Heterozygous DNA and homozygous DNA samples for each SNP were used.

Optimal quality control (QC) parameters considered were: 1) more than 100–200 of merged events in plasma samples, and 2) more than 10000 droplets per replicate.

### Digital PCR data interpretation

Data were analyzed using the QuantaSoft software (Bio-Rad, Pleasanton, CA). CffDNA percentage was determined based on the ratio between alleles when these were different, and the value was calculated by the software. For the analysis of maternally inherited fetal alleles, RMD was calculated based on the z-score empirical values and three different categories were defined: balanced allelic ratio, imbalanced allelic ratio, and inconclusive or grey zone (see S1 File).

#### Confirmation of the presence of ccffDNA in maternal plasma samples

Those cases where a) an exclusively paternal inherited fetal allele was detected, or b) an allelic imbalance between allele 1 and allele 2 was observed in the maternal plasma, the presence of ccffDNA was assumed and no further confirmation analysis of the presence of fetal DNA was needed.

When a balanced allelic ratio is observed, fetal genotype is assumed to be identical to the maternal one. Nevertheless, confirmation of the presence of fetal DNA in the maternal plasma is recommended to avoid any possible misdiagnosis due to the lack of fetal DNA. As detailed below, in the present study two different strategies were used according to fetal gender which was determined following the previously described protocol [5].

In case of male pregnancies: *SRY* and *GAPDH* genes were coamplified simultaneously by a multiplex digital PCR assay. First, a temperature gradient was performed in order to determine the optimal annealing temperature for coamplification of both amplicons (Fig 2). Then, digital PCR amplification in DNA from maternal plasma was carried out following the same protocol as described for the SNP analysis.

In case of female pregnancies: DNA extracted from maternal plasma was digested with the methylation-sensitive restriction enzyme BstUI (New England Biolabs, Inc., USA) at 60°C for 16 h in a final reaction volume of 50 μl containing 2 μl of BstUI, 5 μl 1X NE Buffer, and 43 μl of DNA. A subsequent temperature gradient was performed to set up the optimal annealing temperature for coamplification of *RASSF1A* and *GAPDH* genes by multiplex digital PCR assay (Fig 2). Finally, digital PCR amplification in DNA from maternal plasma was carried out.

## Results

### SNP Genotyping Results

After genotype analysis of 13 couples for six autosomal SNPs and five couples for one SNP on Chromosome X, a total of 55 SNPs were considered informative for the purpose of the study (S1 Table).

All NIPD results were compared with conventional PD results (S1 Table).

*Autosomal SNPs (50/55):*

- Nineteen out of 50 cases were considered for analysis of paternally inherited alleles not present in the maternal genome.

In 9 out of 19 cases, the paternal allele was detected.

One case was not considered for further analysis since it did not reach the optimal QC parameters.

In the remaining nine, no paternal alleles were detected in maternal plasma and further confirmation of the presence of fetal DNA was carried out by: 1) imbalanced allelic ratio for another SNP (5/9); 2) analysis of the *SRY* gene (1/9); and 3) analysis of RASS*F1A* gene (3/9).

**Fig 3:**
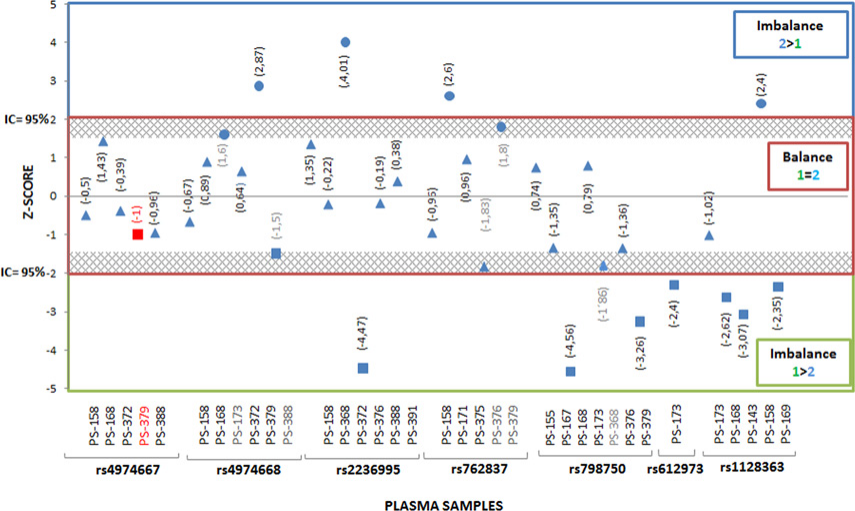
Results of Z-Score values. This figure shows the results of Z-Score values calculated in all thirty-six cases considered for Relative Mutation Dosage (RMD) to establish the fetal genotyping in maternal plasma samples. Balance and Imbalance allelic ratio was used to ascertain the fetal genotype. Blue circle = Homozygous fetus for Allele 2 (2>1); Blue triangle = Heterozygous Allele 1/ Allele 2 fetus (2 = 1); Blue square = Homozygous fetus for Allele 1 (1>2)and Red square = False Positive.

- Thirty-one out of 50 cases were considered for RMD. Balanced or imbalanced allelic ratio was used to ascertain the fetal genotype (Fig 3).

Seven out of 31 cases showed an imbalanced allelic ratio between alleles 1 and 2. Of those: 1) four exhibited overrepresentation of allele 1, and 2) three displayed overrepresentation of allele 2.

In 23 out of 31 cases, a balanced allelic ratio between alleles 1 and 2 was observed. In these cases, confirmation of the presence of fetal DNA was done by: a) an allelic imbalance ratio in another SNP included in the study (15/22); b) detection of the *SRY* gene (4/22); and c) detection of the *RASSF1A* gene (4/22).

One case (1/31) was not considered for further analysis since QC parameters were sub-optimal.

Plasma sample results were compared with the fetal genotype obtained in CVS or AF (S1 Table): a) in 24/30 cases, plasma samples results were in agreement with fetal genotype; b) 1/30 showed a discrepancy between the result obtained in the plasma sample and that obtained by conventional PD and c) 5/30 were within the grey zone and considered unconclusive.

*X-linked SNPs (5/55)*: All cases were considered for RMD.

- Female cases (3/5): two cases showed an imbalanced allelic ratio and the remaining one showed a balanced ratio (Fig 3). In the latter, confirmation of presence of ccffDNA in the plasma sample was performed by the detection of the *RASSF1A* gene.
- Male cases (2/5): in male fetuses, an imbalanced ratio allelic for the maternal X chromosome allele is expected. One case was hemizygous for allele 1 and the other one was hemizygous for allele 2 (Fig 3).

#### Confirmation and quantification of ccffDNA in maternal plasma samples

Paternal allele detection cases revealed that the percentage of fetal DNA ranged from 5% to 21% (median, 13%).

- *SRY/GAPDH* strategy was used in one maternal plasma sample, revealing a percentage of 15.8%.
- *RASSF1A/GAPDH* strategy was used in three maternal plasma samples, revealing a percentage of 2.5%, 4.4%, and 10.8%.

In order to estimate the reproducibility of the assay, the Coefficient of Variation (CV) was calculated. Since the software analysis of the ddPCR applies a Poisson statistics, CV is calculated as √N/N where N = number of target molecules.

The % CV ranged from 4.7–10%.

## Discussion

To date, NIPD study of *de novo* or paternally inherited monogenic disorders has been achieved using routine diagnostic methods. The detection or non-detection of these alleles not present in the mother can be associated with a fetal genotype, thus allowing diagnosis [19– 21].

A wide range of molecular techniques such as restriction analysis, automated sequencing, QF-PCR, high resolution melting, and minisequencing [22] have proven potential for NIPD detection of paternally inherited fetal alleles. Previously reported examples of these achievements include NIPD of pathologies like achondroplasia [23, 24], myotonic dystrophy [25], cystic fibrosis [16, 26–28], or Huntington disease [29–31], among others. Recently, NGS panels for NIPD of different mutations associated with cystic fibrosis and skeletal dysplasias are available in clinical practice in the UK [7, 8]. Nevertheless, they are still limited to the study of *de novo* or paternally inherited fetal mutations.

As for the non-invasive diagnosis of maternally inherited disorders, the coexistence of maternal and fetal DNA in the sample makes this diagnosis more challenging, and therefore this study is currently not being considered during genetic counseling of couples at risk of a Mendelian disorder. However, this scenario is changing thanks to new counting technologies (NGS and digital PCR) that make it possible to perform NIPD of both paternally and maternally inherited fetal alleles. Digital PCR allows a precise allele quantification and comparison between allelic dosages (RMD) that can be used for fetal genotyping [17].

In this work, the potential of Droplet Digital PCR (ddPCR) technology has been evaluated for non-invasive prenatal genotyping of fetal alleles independently of parental origin. In order to evaluate the clinical potential of digital PCR for NIPD of monogenic disorders, all of the different possible PD scenarios that geneticists can encounter in clinical practice have been mimicked by an SNP genotyping approach.

In the case of the less challenging scenario, detection of paternally inherited alleles, ddPCR showed 100% accuracy, sensitivity, Positive Predictive Value (PPV), and Negative Predictive Value (NPV). Therefore, this strategy has been shown to be effective for this aim and can be considered as an alternative for the techniques already in use.

**Fig 4:**
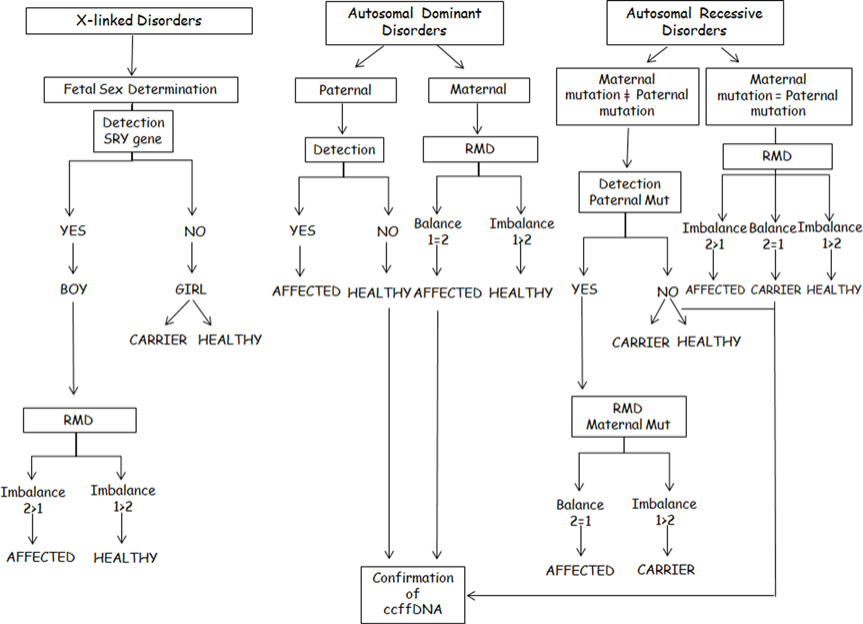
Clinical interpretation algorithm where Allele 1 (1) represents the maternal healthy allele and Allele 2 (2) represents the maternal mutated allele.

By the use of ddPCR not only the paternal alleles can be detected in maternal plasma [32]; its added value is the possibility to study those maternal alleles inherited by the fetus [33]. Although previous experience of this technology for the maternally inherited fetal alleles is limited, some studies have demonstrated the proof of principle and the potential the technology has; these studies include the analysis of single gene disorders where both parents carry the same mutation [34] or dominant disorders where the mother is the carrier [35]. This fact brings the field of NIPD a step further, broadening its scope to the study of any Mendelian disorder independently of its parental inheritance (Fig 1). This genotyping approach is not based on presence/absence criteria but on an accurate relative allelic ratio measurement; hence a balanced or imbalanced allelic ratio is associated with a fetal genotype. A balanced allelic ratio would indicate a fetus with the same genotype as the mother (heterozygous fetus). On the contrary, an imbalanced allelic ratio would be associated with a homozygous fetus for the overrepresented allele. The clinical interpretation algorithm of the balance/imbalance would depend on the inheritance pattern of the disease (Fig 4). Hence, in case of a maternal dominant disease, a balanced allelic ratio between Wild-Type (WT) and mutant alleles would be associated with an affected fetus. On the other hand, an imbalanced allelic ratio with an overrepresentation of the WT allele would be associated with a healthy fetus. In case of a recessive disease where the parents are carriers of the same mutation, an allelic imbalance would be associated with a homozygous fetus and the overrepresented allele would define the healthy (WT) or affected (MUT) status of the fetus. In case of a balanced allelic ratio, a carrier fetus for the maternal mutation is determined.

A special situation is the clinical interpretation of X-linked disorders in which fetal sex needs to be taken into consideration together with the balanced/imbalaced allelic ratio. Fetal sex assessment in maternal blood by means of RT-PCR allows the reduction of invasive testing to male foetuses [36, 37]. The incorporation of ddPCR allows the completion of this analysis by determining which maternal X chromosome has been inherited by the fetus, since it will be overrepresented in the RMD analysis.

In the present study, ddPCR for NIPD of maternally inherited fetal alleles has shown an accuracy of 96%, a NPV of 100% and a PPV of 97% since a FP case was detected.

In 24 out of 31 cases, the results derived from the RMD approach in maternal blood were consistent with fetal genotypes. One was discordant with the fetal genotype (a FP result). Five out of 31 cases were considered as inconclusive since their Z-score values were within the grey zone (see S1 File). One case had not optimal QC parameters; therefore, no further analysis was performed. The cases in which QC parameters were not optimal or in which no conclusive results were obtained would require the collection of a new sample. However, since this was a retrospective study, we had a limited amount of maternal plasma sample and no further testing was possible. Our NIPD protocol calls for the collection of two or even three plasma samples before a result is reported. This protocol will also apply to ddPCR studies, thus reducing the possible drawbacks. The FP (sample 379 for rs4974667) was because a balanced allelic ratio was observed in the plasma sample when the prenatal sample revealed a homozygous fetus for allele 1. Then, an overrepresentation of allele 1 should have been detected in the maternal plasma sample. The reason for this FP result could have been a cross-hybridization of the Taqman probes (unspecific hybridization of Allele 2 probe), since this phenomenon was also observed in the analysis of the prenatal sample (S1 Table). Presence of unspecific allele 2 amplification events in the plasma sample may have increased the number of events detected for allele 2 reducing the allelic imbalance.

As previously described, a balanced RMD is observed when the mother and fetus have the same genotype. However, the lack of ccffDNA in the sample could lead to the same result, possibly leading to a misdiagnosis. The high sensitivity that the ddPCR technology has (detection of rare events down to 0,1%, data not shown) allows to analyze maternal plasma samples with low ccffDNA percentages (S1 Table). However, in order to discard the risk of a misdiagnosis because of a false balanced ratio, it is essential perform a complementary analysis to confirm the presence of ccffDNA in the sample.

In this study, 24 samples revealed a balanced allelic ratio in the RMD analysis; therefore, presence of ccffDNA would need further confirmation. Since some of the samples in this study were tested for different SNPs, confirmation could be done indirectly by the analysis of another SNP in which an imbalanced was detected or by other strategies gender dependent. The presence of the *SRY* gene in case of male fetuses confirms the presence of ccffDNA. In case of absence of the *SRY* gene (female fetuses), the *RASSF1A* gene was studied by a methylation-sensitive enzyme digestion.

*RASSF1A* promoter is hypermethylated in fetal DNA and hypomethylated in maternal DNA [38, 39], allowing a differentiation between maternal and fetal DNA and thus detecting the presence of fetal DNA independently of its sex. Three female plasma samples required this ccffDNA confirmation strategy revealing a range of ccffDNA percentage comprised between 2.5–10.2% at 12–13 weeks of gestation. It has been described that quantification based on *RASSF1A* analysis can lead to an underestimation of the ccffDNA percentage due to an incomplete hypermethylation of ccffDNA, which may result in the digestion of hypomethylated fetal fragments [40]. As a consequence, *RASSF1A* can be considered a good tool for the confirmation of the presence of ccffDNA in the sample, independently of the fetal sex; however, it is not the most suitable method for the measurement of fetal DNA fraction.

As shown in this work, ddPCR allows for precise quantification of fetal alleles and a prenatal clinical correlation can be done on the basis of the results. NIPD based on NGS panels covers the most frequent mutations associated to a specific pathology. This strategy is a versatile approach since it can be applied to many different pregnancies. However, rare diseases are frequently caused by mutations with a very low frequency and in many cases are limited to a family pedigree (i.e., metabolic diseases) [20] and therefore may not be included in the panels. In this situation, individualized diagnostic approaches are needed, and ddPCR would play a role. In conventional prenatal diagnosis nowadays, NIPD units for rare diseases would require the availability of different diagnostic tools to be applied in the many different situations that they may face.

To conclude, this work demonstrates the potential of ddPCR for NIPD fetal genotyping independently of the inheritance pattern and the parental origin of the alleles. This technology has been shown to be precise, sensitive, rapid, and easy to interpret without a need for complex bioinformatic tools.

## Supporting Information

S1 File From RMD to fetal genotype. Z-score values will be applied to establish the fetal genotyping in maternal plasma samples for the study of maternal disorders (heterozygous mothers).
(DOCX)

S1 Table SNPs genotyping results. SNP genotyping results from plasma samples studied in 15 couples and 7 SNPs and the concordance with the conventional Prenatal Diagnosis results. Weeks of gestation (w) for each plasma sample studied are specified. NIPD = Non Invasive Prenatal Diagnosis; PD = Prenatal Diagnosis; CFG = Concordant with Fetal Genotype; NQC = Quality Control Not passed; FP = False Positive; GZ = Grey Zone. Blue background = paternal dominant disorders; Red background = maternal dominant disorders; Yellow background = recessive disorders where parents are carriers of the same mutation and Green background = X-linked disorders.
(TIF)
