# Supplementary material for: Fetal Genotyping in Maternal Blood by Digital PCR: Towards NIPD of Monogenic Disorders Independently of Parental Origin
Source: PLoS One. 2016 Apr 14;11(4):e0153258. doi: 10.1371/journal.pone.0153258 (PMC4831728; doi:10.1371/journal.pone.0153258)
Supplement: S1 File — Z-score values will be applied to establish the fetal genotyping in maternal plasma samples for the study of maternal disorders (heterozygous mothers). (DOCX) [file pone.0153258.s001.docx]

**Supporting information:**

**S1 File. From RMD to fetal genotype.**

Z-score is a measurement that indicates how many standard deviations an element is from the mean.

Z-score can be calculated from the following formula:


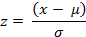


Where z is the Z-score, x is the value of the element, μ is the population mean, and σ is the population standard deviation.

If the fetus is heterozygous:

$x=N_{2}-N_{1}$

$${(N}_{2}=number of counts for allele 2)$$

$${(N}_{1}=number of counts for allele 1)$$

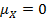


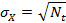


So the Z-score is:

$$z= \frac{(x-\mu_{x})}{\sigma_{x}}= \frac{N_{2}- N_{1}}{\sqrt{N_{t}}}$$

$$N_{t}= N_{2}+ N_{1}$$

Z-score values will be applied to establish the fetal genotyping in maternal plasma samples for the study of maternal disorders (heterozygous mothers).

1. **Maternally inherited autosomal SNPs**
2. *Heterozygous 1/2 fetus*: when counts for allele 2 and counts for allele 1 are nearly equal (range of Z-score comprised between -2 and 2 values). A grey zone has been established (range of Z-score between -1.5/-2 and 1.5/2) when a new sample is needed, because of an incongruent result.
3. *Homozygous 2/2 fetus*: when counts for allele 2 are overrepresented with respect to allele 1. For Z-score values above 2 (>2) with a confidence interval (CI) of 95% and for Z-score values above 3 (>3) a CI of 99%.
4. *Homozygous 1/1 fetus*: when counts for allele 1 are overrepresented with respect to allele 2. For Z-score values below -2 (<-2) with a CI of 95% and for z-score values below -3 (<-3) a CI of 99%.
5. **X-linked SNP**

a) *For female fetuses*; same interpretation as an autosomal disease.

b) *For male fetuses*; the presence of a unique X chromosome implies that only one fetal allele contributes to the imbalance. Therefore, an overrepresentation of the allele 2 or the allele 1 (the one inherited from the mother) is expected.

In case of a heterozygous fetus (same genotype as the mother), it could be due to a) a real heterozygousfetus, or b) a failure to detect ccffDNA. In order to discard the possibility of a failure of detection, a strategy to confirm the presence of ccffDNA is recommended.
