# Supplementary figures and images for: Fetal Genotyping in Maternal Blood by Digital PCR: Towards NIPD of Monogenic Disorders Independently of Parental Origin
Source: PLoS One. 2016 Apr 14;11(4):e0153258. doi: 10.1371/journal.pone.0153258 (PMC4831728; doi:10.1371/journal.pone.0153258)

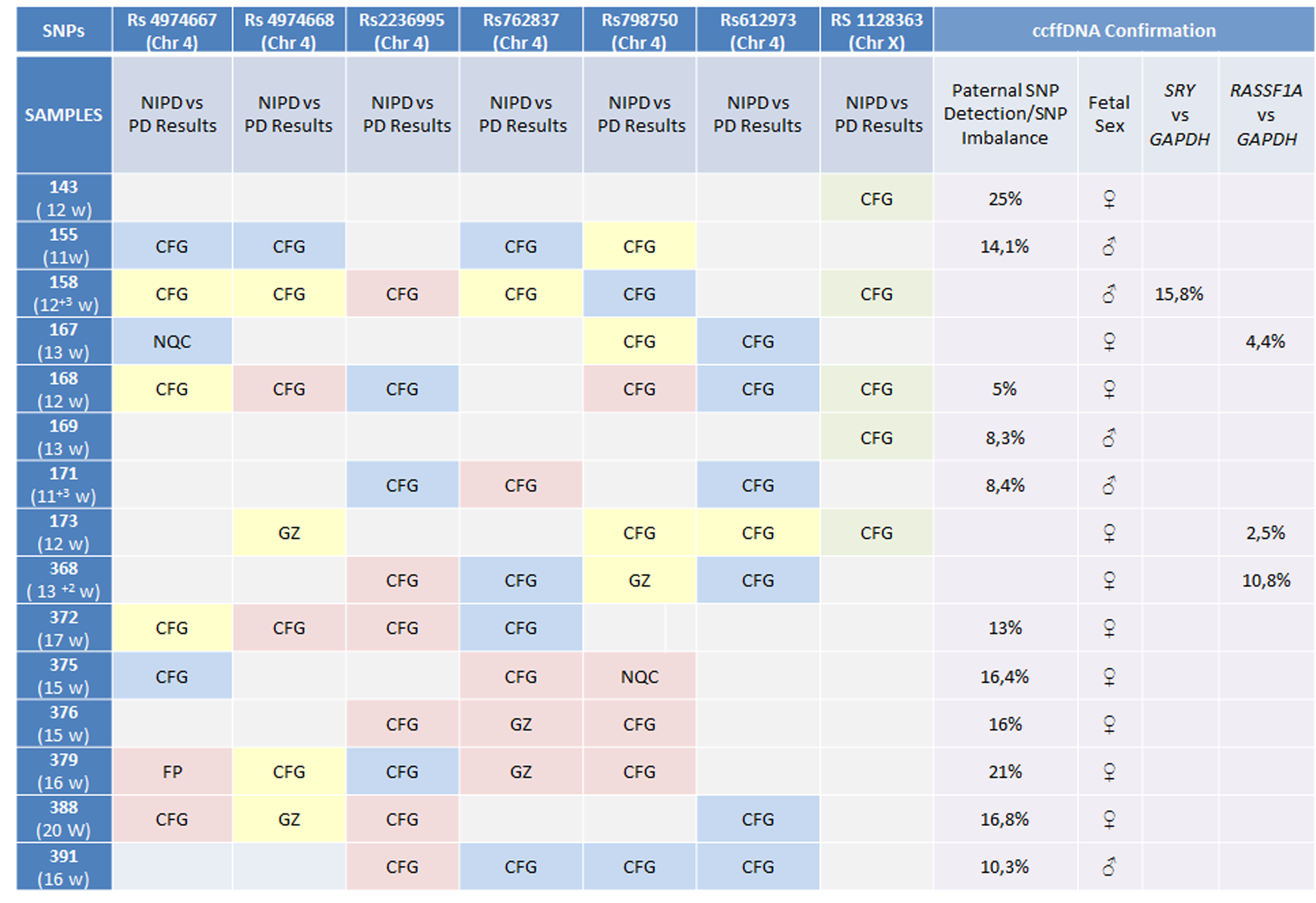

Supplement: S1 Table — SNP genotyping results from plasma samples studied in 15 couples and 7 SNPs and the concordance with the conventional Prenatal Diagnosis results. Weeks of gestation (w) for each plasma sample studied are specified. NIPD = Non Invasive Prenatal Diagnosis; PD = Prenatal Diagnosis; CFG = Concordant with Fetal Genotype; NQC = Quality Control Not passed; FP = False Positive; GZ = Grey Zone. Blue background = paternal dominant disorders; Red background = maternal dominant disorders; Yellow background = recessive disorders where parents are carriers of the same mutation and Green background = X-linked disorders. (TIF) [file pone.0153258.s002.tif]
